# Supplementary material for: The ChiS-Family DNA-Binding Domain Contains a Cryptic Helix-Turn-Helix Variant
Source: mBio. 2021 Mar 16;12(2):e03287-20. doi: 10.1128/mBio.03287-20 (PMC8092284; doi:10.1128/mBio.03287-20)
Supplement: FIG S1 [file mBio.03287-20-sf001.pdf]

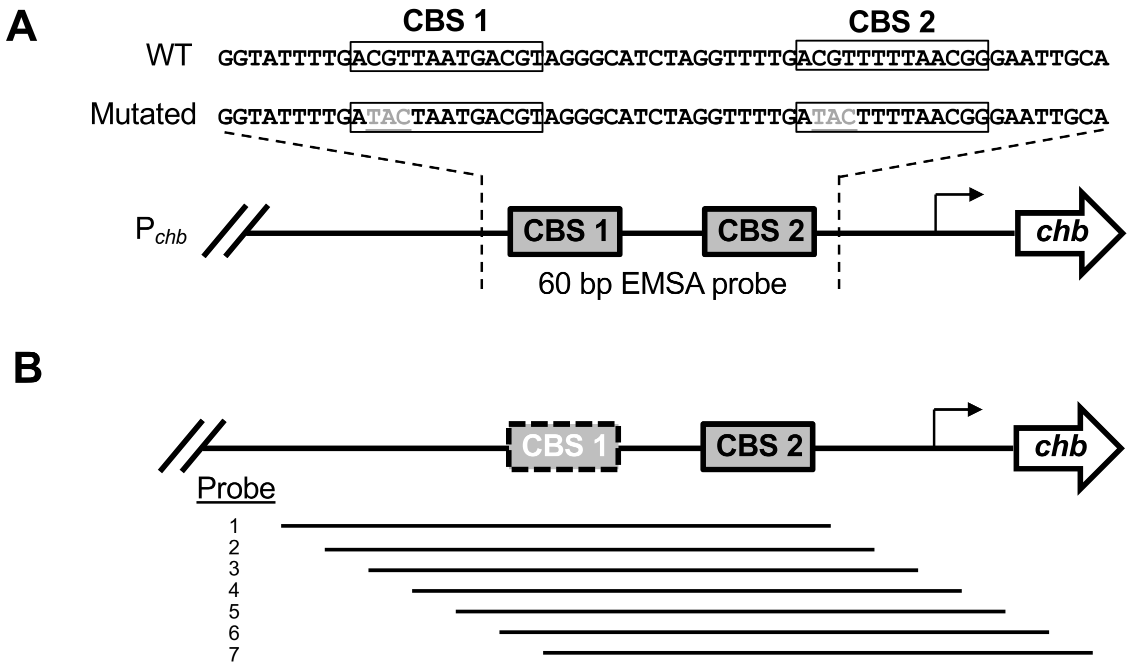

Figure S1. *Diagrams of EMSA probes used in this study.* **A**) Promoter map of *chb* with the region of P_{chb} used for the EMSAs shown in **Figure 1B** indicated. The exact probe sequences are shown above the promoter map. ChiS binding site (CBS) are boxed and the mutations used to disrupt the CBSs are shown in gray and underlined. **B**) Promoter map of *chb* with the region of P_{chb} used for the EMSA shown in **Figure 4B** and **Figure S5**. CBS 1 was mutated (white text, dotted line) in all probes used.
