# Supplementary material for: The ChiS-Family DNA-Binding Domain Contains a Cryptic Helix-Turn-Helix Variant
Source: mBio. 2021 Mar 16;12(2):e03287-20. doi: 10.1128/mBio.03287-20 (PMC8092284; doi:10.1128/mBio.03287-20)
Supplement: FIG S2 [file mBio.03287-20-sf002.pdf]

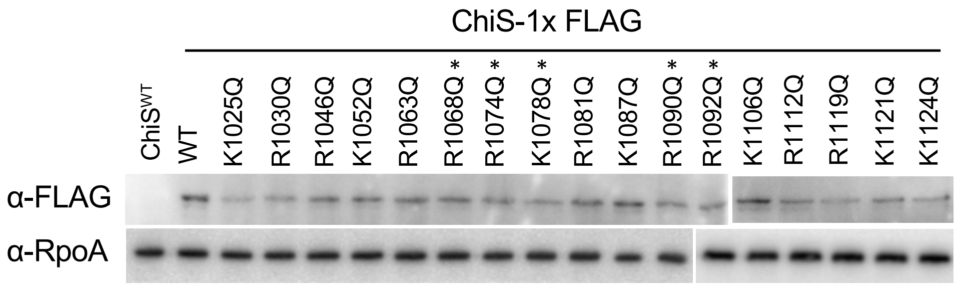

Figure S2. *Mutations to the ChiS DNA binding domain does not prevent expression of ChiS.* Strains expressing the indicated ChiS-FLAG point mutations were assessed for expression by Western blot with anti-FLAG and anti-RpoA (loading control) antibodies. Asterisks above ChiS point mutants indicate the mutations found to be critical for the DNA binding activity of ChiS as shown in **Figure 2**.
