# Supplementary material for: The ChiS-Family DNA-Binding Domain Contains a Cryptic Helix-Turn-Helix Variant
Source: mBio. 2021 Mar 16;12(2):e03287-20. doi: 10.1128/mBio.03287-20 (PMC8092284; doi:10.1128/mBio.03287-20)
Supplement: TABLE S1 [file mBio.03287-20-st001.docx]

| **Table S1. Data collection, processing, and structure refinement**  **A - Data collection and processing**  Values in parentheses are for the outer shell. | |
| --- | --- |
| Diffraction source | Beamline 21ID-F, APS |
| Wavelength (Å) | 0.97872 |
| Temperature (K) | 100 |
| Detector | MAR Mosaic 300 mm CCD |
| Space group | C222_1_ |
| a, b, c (Å) | 51.91, 78.61, 72.37 |
| α, β, γ (°) | 90.00, 90.00, 90.00 |
| Resolution range (Å) | 30.00 - 1.28 (1.30 - 1.28) |
| No. of unique reflections | 38,590 (1,866) |
| Completeness (%) | 99.7 (97.3) |
| Multiplicity | 6.9 (4.6) |
| 〈I/σ(I)〉 | 28.3 (2.3) |
| R_r.i.m._ [^†^](https://www.ncbi.nlm.nih.gov/pmc/articles/PMC5683032/table/table3/?report=objectonly#tfn1) | 0.032 (0.386) |
| CC_1/2_[^††^](https://www.ncbi.nlm.nih.gov/pmc/articles/PMC5683032/table/table3/?report=objectonly#tfn1) | (0.637) |
| Overall B factor from Wilson plot (Å^2^) | 14.7 |
| ^†^Estimated R_r.i.m._ = R_merge_[N/(N − 1)]^1/2^, where N is the data multiplicity.  [^††^](https://www.ncbi.nlm.nih.gov/pmc/articles/PMC5683032/table/table3/?report=objectonly#tfn1) Pearson’s Correlation Coefficient (Karplus & Diederichs, 2012). | |

| **B - Structure refinement**  Values in parentheses are for the outer shell. | |
| --- | --- |
| Resolution range (Å) | 25.97 - 1.28 (1.31 - 1.28) |
| Completeness (%) | 99.7 (98.3) |
| No. of reflections, working set | 36,482 (2,634) |
| No. of reflections, test set | 1,885 (138) |
| Final R _work_ | 0.144 (0.214) |
| Final R _free_ | 0.180 (0.238) |
| No. of non-H atoms | |
| Protein | 989 |
| Ligand | 33 |
| Water | 200 |
| Total | 1,222 |
| R.m.s. deviations | |
| Bonds (Å) | 0.005 |
| Angles (°) | 1.217 |
| Average B factors (Å^2^) |  |
| Protein | 17.8 |
| Ligand | 31.6 |
| Water | 31.3 |
| Ramachandran plot | |
| Favored regions (%) | 99.0 |
| Additionally allowed (%) | 1.0 |
| Outliers (%) | 0.0 |
