# Supplementary material for: The ChiS-Family DNA-Binding Domain Contains a Cryptic Helix-Turn-Helix Variant
Source: mBio. 2021 Mar 16;12(2):e03287-20. doi: 10.1128/mBio.03287-20 (PMC8092284; doi:10.1128/mBio.03287-20)
Supplement: FIG S3 [file mBio.03287-20-sf003.pdf]

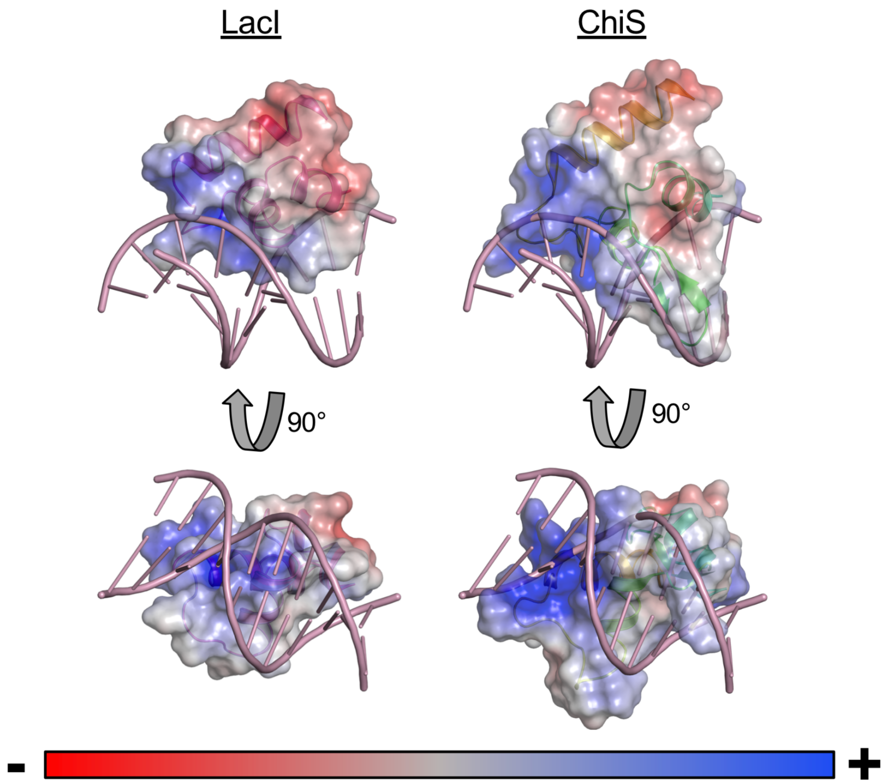

Figure S3. *The electrostatic surface pattern of the LacI helix-turn-helix and ChiS helix-sheet-helix are similar.* Electrostatic maps of the LacI helix-turn-helix DNA-bound structure and the ChiS helix-sheet-helix DNA-bound model. The DNA shown is from the LacI structure, and the ChiS DBD was modeled onto DNA by alignment to LacI as shown in **Figure 3C**. Regions of the protein surface colored in red are negatively charged, while those shown in blue are positively charged.
