# Supplementary material for: The ChiS-Family DNA-Binding Domain Contains a Cryptic Helix-Turn-Helix Variant
Source: mBio. 2021 Mar 16;12(2):e03287-20. doi: 10.1128/mBio.03287-20 (PMC8092284; doi:10.1128/mBio.03287-20)
Supplement: FIG S4 [file mBio.03287-20-sf004.pdf]

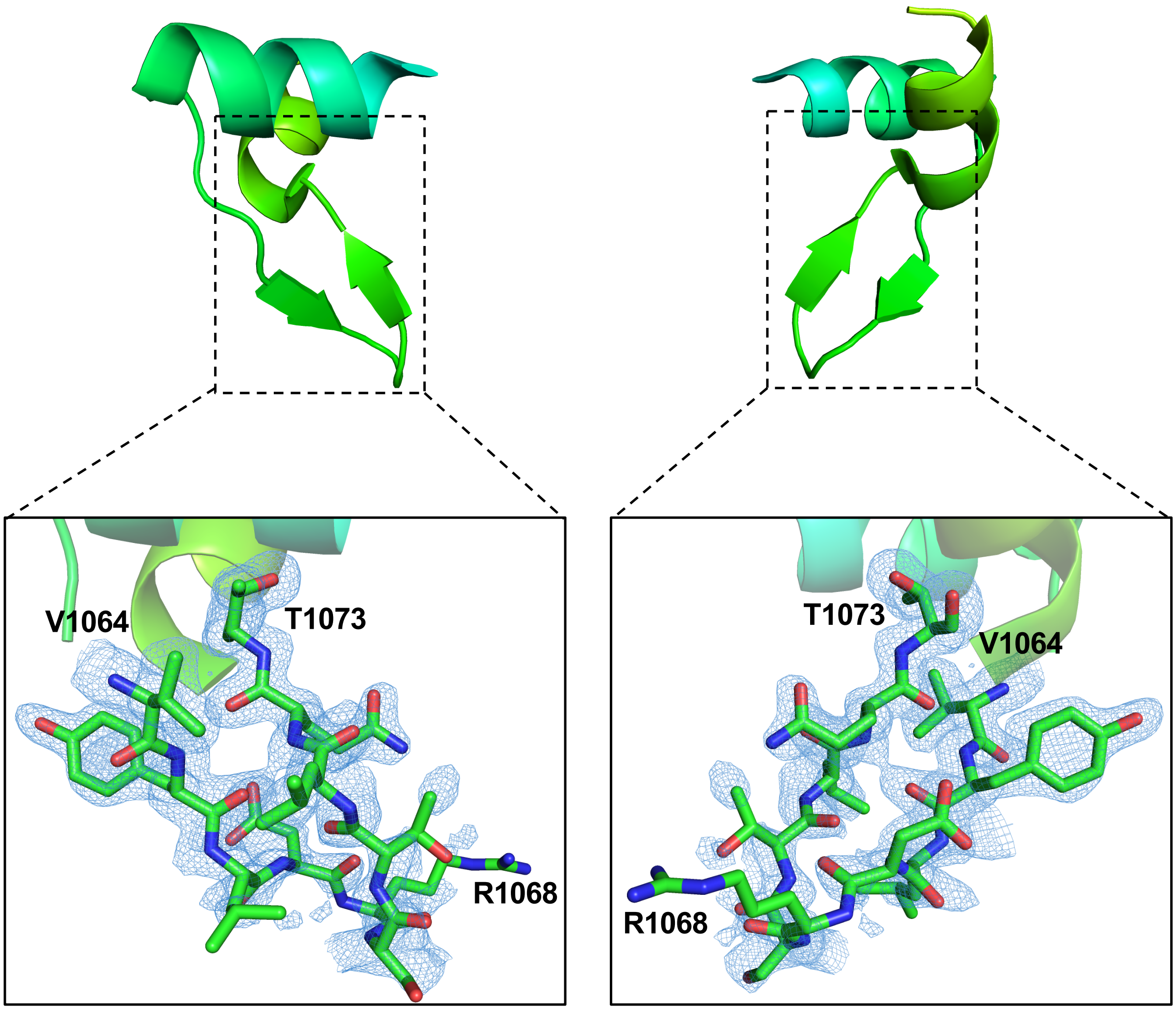


**Figure S4.** *Electron density map of the β-sheet insertion within the helix-sheet-helix.* An omit $F_o$ - $F_c$ map contoured at the 2.0 sigma level (omitted V1064-T1073) was generated and mapped onto the β-sheet insertion of the ChiS helix-sheet-helix. The map reveals that despite the high B-factor in this region, the structure modeled within the antiparallel beta strands are strongly supported by the data collected. The modeled side chains of the residues in the turn of the beta-turn-beta insertion, however, are less clearly resolved, which may be due to the flexibility of this region.
