# Supplementary material for: The ChiS-Family DNA-Binding Domain Contains a Cryptic Helix-Turn-Helix Variant
Source: mBio. 2021 Mar 16;12(2):e03287-20. doi: 10.1128/mBio.03287-20 (PMC8092284; doi:10.1128/mBio.03287-20)
Supplement: FIG S5 [file mBio.03287-20-sf005.pdf]

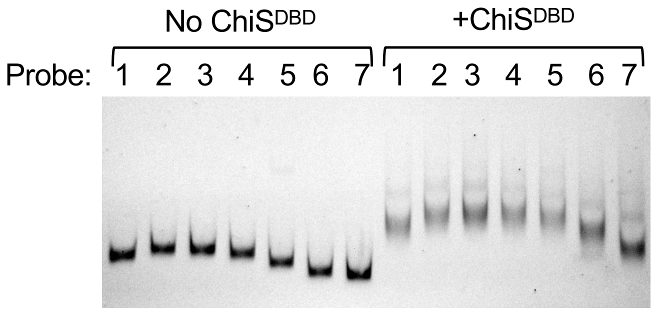

Figure S5. *ChiS protein does not further bend the P_{chb} promoter.* The probes shown in **Figure S1B** were incubated in the absence (No ChiS^{DBD}) or presence (+ChiS^{DBD}) of 400 nM ChiS^{DBD}.
