# Supplementary material for: The ChiS-Family DNA-Binding Domain Contains a Cryptic Helix-Turn-Helix Variant
Source: mBio. 2021 Mar 16;12(2):e03287-20. doi: 10.1128/mBio.03287-20 (PMC8092284; doi:10.1128/mBio.03287-20)
Supplement: FIG S6 [file mBio.03287-20-sf006.pdf]

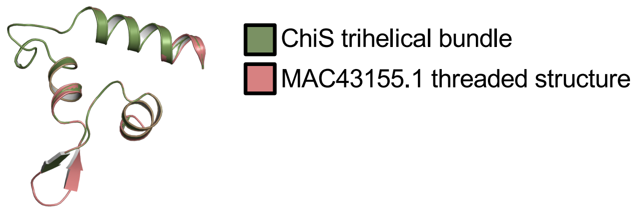

Figure S6. *The most dissimilar ChiS DBD homolog threads onto the trihelical bundle of the ChiS DBD structure.* The sequence of the ChiS-family DBD from MAC43155.1 was threaded onto the crystal structure of the ChiS DBD using Phyre2 (9). Alignment of alpha carbons gave an RMSD of 0.002.
