# Supplementary material for: The ChiS-Family DNA-Binding Domain Contains a Cryptic Helix-Turn-Helix Variant
Source: mBio. 2021 Mar 16;12(2):e03287-20. doi: 10.1128/mBio.03287-20 (PMC8092284; doi:10.1128/mBio.03287-20)
Supplement: TABLE S2 [file mBio.03287-20-st002.docx]

| **Strain (Reference)**  **Table S2.** *Strains used in this study.* | **Reference in Manuscript** | **Genotype** |
| --- | --- | --- |
| SAD 030 | Parent strain for all other *V. cholerae* strains in this study | *V. cholerae* E7946 WT Sm^R^ |
| SAD 2706 | Figure 2 ChIP WT & Figure S1 WT | ∆*lacZ:*:P*_chb_*-GFP, Kan^R^; ∆*chiS/cbp*::Carb^R^; ∆VCA0692::ChiS 1x FLAG @ E566, TmR |
| SAD 3006 | Figure 2 ChIP K1025Q & Figure S1 K1025Q | ∆*lacZ*::P*_chb_*-GFP, Kan^R^; ∆*chiS/cbp*::Carb^R^; ∆VCA0692::ChiS 1x FLAG @ E566 K1025Q, Tm^R^ |
| SAD 3007 | Figure 2 ChIP R1030Q & Figure S1 R1030Q | ∆*lacZ*::P*_chb_*-GFP, Kan^R^; ∆*chiS/cbp*::Carb^R^; ∆VCA0692::ChiS 1x FLAG @ E566 R1030Q, Tm^R^ |
| SAD 3008 | Figure 2 ChIP R1046Q & Figure S1 R1046Q | ∆*lacZ*::P*_chb_*-GFP, Kan^R^; ∆*chiS/cbp*::Carb^R^; ∆VCA0692::ChiS 1x FLAG @ E566 R1046Q, Tm^R^ |
| SAD 3009 | Figure 2 ChIP K1052Q & Figure S1 K1052Q | ∆*lacZ*::P*_chb_*-GFP, Kan^R^; ∆*chiS/cbp*::Carb^R^; ∆VCA0692::ChiS 1x FLAG @ E566 K1052Q, Tm^R^ |
| SAD 3010 | Figure 2 ChIP R1063Q & Figure S1 R1063Q | ∆*lacZ*::P*_chb_*-GFP, Kan^R^; ∆*chiS/cbp*::Carb^R^; ∆VCA0692::ChiS 1x FLAG @ E566 R1063Q, Tm^R^ |
| SAD 3011 | Figure 2 ChIP R1068Q & Figure S1 R1068Q | ∆*lacZ*::P*_chb_*-GFP, Kan^R^; ∆*chiS/cbp*::Carb^R^; ∆VCA0692::ChiS 1x FLAG @ E566 R1068Q, Tm^R^ |
| SAD 3012 | Figure 2 ChIP R1074Q & Figure S1 R1074Q | ∆*lacZ*::P*_chb_*-GFP, Kan^R^; ∆*chiS/cbp*::Carb^R^; ∆VCA0692::ChiS 1x FLAG @ E566 R1074Q, Tm^R^ |
| SAD 3013 | Figure 2 ChIP K1078Q & Figure S1 K1078Q | ∆*lacZ*::P*_chb_*-GFP, Kan^R^; ∆*chiS/cbp*::Carb^R^; ∆VCA0692::ChiS 1x FLAG @ E566 K1078Q, Tm^R^ |
| SAD 3014 | Figure 2 ChIP R1081Q & Figure S1 R1081Q | ∆*lacZ*::P*_chb_*-GFP, Kan^R^; ∆*chiS/cbp*::Carb^R^; ∆VCA0692::ChiS 1x FLAG @ E566 R1081Q, Tm^R^ |
| SAD 3015 | Figure 2 ChIP K1087Q & Figure S1 K1087Q | ∆*lacZ*::P*_chb_*-GFP, Kan^R^; ∆*chiS/cbp*::Carb^R^; ∆VCA0692::ChiS 1x FLAG @ E566 K1087Q, Tm^R^ |
| SAD 3016 | Figure 2 ChIP R1090Q & Figure S1 R1090Q | ∆*lacZ*::P*_chb_*-GFP, Kan^R^; ∆*chiS/cbp*::Carb^R^; ∆VCA0692::ChiS 1x FLAG @ E566 R1090Q, Tm^R^ |
| SAD 3017 | Figure 2 ChIP R1092Q & Figure S1 R1092Q | ∆*lacZ*::P*_chb_*-GFP, Kan^R^; ∆*chiS/cbp*::Carb^R^; ∆VCA0692::ChiS 1x FLAG @ E566 R1092Q, Tm^R^ |
| SAD 3018 | Figure 2 ChIP K1106Q & Figure S1 K1106Q | ∆*lacZ*::P*_chb_*-GFP, Kan^R^; ∆*chiS/cbp*::Carb^R^; ∆VCA0692::ChiS 1x FLAG @ E566 K1106Q, Tm^R^ |
| SAD 3019 | Figure 2 ChIP R1112Q & Figure S1 R1112Q | ∆*lacZ*::P*_chb_*-GFP, Kan^R^; ∆*chiS/cbp*::Carb^R^; ∆VCA0692::ChiS 1x FLAG @ E566 R1112Q, Tm^R^ |
| SAD 3020 | Figure 2 ChIP R1119Q & Figure S1 R1119Q | ∆*lacZ*::P*_chb_*-GFP, Kan^R^; ∆*chiS/cbp*::Carb^R^; ∆VCA0692::ChiS 1x FLAG @ E566 R1119Q, Tm^R^ |
| SAD 3021 | Figure 2 ChIP K1121Q & Figure S1 K1121Q | ∆*lacZ*::P*_chb_*-GFP, Kan^R^; ∆*chiS/cbp*::Carb^R^; ∆VCA0692::ChiS 1x FLAG @ E566 K1121Q, Tm^R^ |
| SAD 3022 | Figure 2 ChIP K1124Q & Figure S1 K1124Q | ∆*lacZ*::P*_chb_*-GFP, Kan^R^; ∆*chiS/cbp*::Carb^R^; ∆VCA0692::ChiS 1x FLAG @ E566 K1124Q, Tm^R^ |
| SAD 3023 | Figure 2 GFP WT | ∆*lacZ*::P*_chb_*-GFP, Kan^R^; igVCA0265/0266::CBS-mCherry v2, Cm^R^; ∆chiS/cbp::Carb^R^; ∆VCA0692::ChiS, Tm^R^ |
| SAD 3024 | Figure 2 GFP K1025Q | ∆*lacZ*::P*_chb_*-GFP, Kan^R^; igVCA0265/0266::CBS-mCherry v2, Cm^R^; ∆chiS/cbp::Carb^R^; ∆VCA0692::ChiS K1025Q, Tm^R^ |
| SAD 3025 | Figure 2 GFP R1030Q | ∆*lacZ*::P*_chb_*-GFP, Kan^R^; igVCA0265/0266::CBS-mCherry v2, Cm^R^; ∆chiS/cbp::Carb^R^; ∆VCA0692::ChiS R1030Q, Tm^R^ |
| SAD 3026 | Figure 2 GFP R1046Q | ∆*lacZ*::P*_chb_*-GFP, Kan^R^; igVCA0265/0266::CBS-mCherry v2, Cm^R^; ∆chiS/cbp::Carb^R^; ∆VCA0692::ChiS R1046Q, Tm^R^ |
| SAD 3027 | Figure 2 GFP K1052Q | ∆*lacZ*::P*_chb_*-GFP, Kan^R^; igVCA0265/0266::CBS-mCherry v2, Cm^R^; ∆chiS/cbp::Carb^R^; ∆VCA0692::ChiS K1052Q, Tm^R^ |
| SAD 3028 | Figure 2 GFP R1063Q | ∆*lacZ*::P*_chb_*-GFP, Kan^R^; igVCA0265/0266::CBS-mCherry v2, Cm^R^; ∆chiS/cbp::Carb^R^; ∆VCA0692::ChiS R1063Q, Tm^R^ |
| SAD 3029 | Figure 2 GFP R1068Q | ∆*lacZ*::P*_chb_*-GFP, Kan^R^; igVCA0265/0266::CBS-mCherry v2, Cm^R^; ∆chiS/cbp::Carb^R^; ∆VCA0692::ChiS R1068Q, Tm^R^ |
| SAD 3030 | Figure 2 GFP R1074Q | ∆*lacZ*::P*_chb_*-GFP, Kan^R^; igVCA0265/0266::CBS-mCherry v2, Cm^R^; ∆chiS/cbp::Carb^R^; ∆VCA0692::ChiS R1074Q, Tm^R^ |
| SAD 3031 | Figure 2 GFP K1078Q | ∆*lacZ*::P*_chb_*-GFP, Kan^R^; igVCA0265/0266::CBS-mCherry v2, Cm^R^; ∆chiS/cbp::Carb^R^; ∆VCA0692::ChiS K1078Q, Tm^R^ |
| SAD 3032 | Figure 2 GFP R1081Q | ∆*lacZ*::P*_chb_*-GFP, Kan^R^; igVCA0265/0266::CBS-mCherry v2, Cm^R^; ∆chiS/cbp::Carb^R^; ∆VCA0692::ChiS R1081Q, Tm^R^ |
| SAD 3033 | Figure 2 GFP K1087Q | ∆*lacZ*::P*_chb_*-GFP, Kan^R^; igVCA0265/0266::CBS-mCherry v2, Cm^R^; ∆chiS/cbp::Carb^R^; ∆VCA0692::ChiS K1087Q, Tm^R^ |
| SAD 3034 | Figure 2 GFP R1090Q | ∆*lacZ*::P*_chb_*-GFP, Kan^R^; igVCA0265/0266::CBS-mCherry v2, Cm^R^; ∆chiS/cbp::Carb^R^; ∆VCA0692::ChiS R1090Q, Tm^R^ |
| SAD 3035 | Figure 2 GFP R1092Q | ∆*lacZ*::P*_chb_*-GFP, Kan^R^; igVCA0265/0266::CBS-mCherry v2, Cm^R^; ∆chiS/cbp::Carb^R^; ∆VCA0692::ChiS R1092Q, Tm^R^ |
| SAD 3036 | Figure 2 GFP K1106Q | ∆*lacZ*::P*_chb_*-GFP, Kan^R^; igVCA0265/0266::CBS-mCherry v2, Cm^R^; ∆chiS/cbp::Carb^R^; ∆VCA0692::ChiS K1106Q, Tm^R^ |
| SAD 3037 | Figure 2 GFP R1112Q | ∆*lacZ*::P*_chb_*-GFP, Kan^R^; igVCA0265/0266::CBS-mCherry v2, Cm^R^; ∆chiS/cbp::Carb^R^; ∆VCA0692::ChiS R1112Q, Tm^R^ |
| SAD 3038 | Figure 2 GFP R1119Q | ∆*lacZ*::P*_chb_*-GFP, Kan^R^; igVCA0265/0266::CBS-mCherry v2, Cm^R^; ∆chiS/cbp::Carb^R^; ∆VCA0692::ChiS R1119Q, Tm^R^ |
| SAD 3039 | Figure 2 GFP K1121Q | ∆*lacZ*::P*_chb_*-GFP, Kan^R^; igVCA0265/0266::CBS-mCherry v2, Cm^R^; ∆chiS/cbp::Carb^R^; ∆VCA0692::ChiS K1121Q, Tm^R^ |
| SAD 3040 | Figure 2 GFP K1124Q | ∆*lacZ*::P*_chb_*-GFP, Kan^R^; igVCA0265/0266::CBS-mCherry v2, Cm^R^; ∆chiS/cbp::Carb^R^; ∆VCA0692::ChiS K1124Q, Tm^R^ |
| SAD 2675 | Figure S2 ChiS^WT^ No FLAG tag | ∆*lacZ*:P*_chb_*-GFP, Kan^R^; ∆*chiS/cbp*::Carb^R^; ∆VCA0692::ChiS, Tm^R^ |
