# Supplementary material for: The ChiS-Family DNA-Binding Domain Contains a Cryptic Helix-Turn-Helix Variant
Source: mBio. 2021 Mar 16;12(2):e03287-20. doi: 10.1128/mBio.03287-20 (PMC8092284; doi:10.1128/mBio.03287-20)
Supplement: TABLE S3 [file mBio.03287-20-st003.docx]

| **Primer Name**  **Table S3.** *Primers used in this study.* | **Primer Sequence** | **Description** |
| --- | --- | --- |
| CKP 090 | cggatccGGCTGCTAACAAAG | pHisTev amplify for FastClone F |
| BBC 2359 | atggccctgaaaatacaggttttctatATGGCTGCCGCGCGGCACC | pHisTev amplify for FastClone R |
| CKP 713 | CATatagaaaacctgtattttcagggccatTCGAAACAAGATTTGATGCG | ChiS DBD amplify for FastClone F |
| CKP 484 | CCTTTCGGGCTTTGTTAGCAGCCggatccgTTATTCACTGGTCAGGAGTTTTTGC | ChiS DBD amplify for FastClone R |
| ABD 767 | TTAATTTGGATCCCTGCGACACTC | ∆chiS F1 for Up arm |
| ABD 768 | gtcgacggatccccggaatCAAAAAACGTGAGGAGAATGCC | ∆chiS R1 for Up arm |
| ABD 123 | ATTCCGGGGATCCGTCGAC | Carb^R^ cassette amplify F |
| ABD 124 | TGTAGGCTGGAGCTGCTTC | Carb^R^ cassette amplify R |
| ABD 798 | gaagcagctccagcctacaGTACTGGATCTGAAACCAGTTAAG | ∆cbp F2 for Down arm |
| ABD 799 | GTATTGCGGAATGACCAGCATG | ∆cbp R2 for Down arm |
| ABD 725 | GAAGCAGCTCCAGCCTACA | Detect F for ∆chiS/cbp deletion |
| BBC 082 | gtcgacggatccccggaatCATAACTTACACCTTACTCACCCAG | Detect R for ∆chiS/cbp deletion |
| BBC 832 | GCTTTTTGCTACAACGACCG | ∆VCA0692 Tm^R^ F1 for Up arm |
| BBC 647 | tttttctatttctgaatcgattcatacgaCTCATTAGGCACCCCAGGC | ∆VCA0692 Tm^R^ R1 for Up arm |
| BBC 1889 | tcgtatgaatcgattcagaaatagaaaaaTTTGCCGCTTTTAACGTAAATCAG | ChiS F for Middle arm |
| BBC 577 | tgtaggctggagctgcttcTTATTCACTGGTCAGGAGTTTTTGC | ChiS R for Middle arm |
| BBC 830 | gaagcagctccagcctacaGTTGAGTTGGATGCAGCACC | ∆VCA0692 Tm^R^ F2 for Down arm |
| BBC 834 | CACAATTTCTCGCTTAAAATGTCC | ∆VCA0692 Tm^R^ R2 for Down arm |
| DOG 0718 | gcaggtggagcaggtggaCAACCGGTCTGGGTTTCTG | ChiS internal FLAG at E566 F |
| DOG 0717 | tccaccacttccacctgcTTCTGAGACTTGATTAATAATGCGCAG | ChiS internal FLAG at E566 R |
| BBC 2274 | gcaggtggaagtggtggagattataaggatgacgatgacaaagcaggtggagcaggtgga | 1x FLAG Middle F |
| BBC 2275 | tccacctgctccacctgctttgtcatcgtcatccttataatctccacc acttccacctgc | 1x FLAG Middle R |
| ABD 332 | GGCTGAACGTGGTTGTCGAAAATGAC | ∆lacZ F1 for Up arm |
| BBC 219 | GTTTATTTTTGTCGACTGTACAGCGTTTAAATAGAGGTCGATATTGACCC | ∆lacZ R1 for Up arm |
| BBC 218 | CGCTGTACAGTCGACAAAAATAAAC | Kan^R^ F Middle for GFP reporter |
| BBC 262 | TACCGAGGACGCGAAGCTG | Kan^R^ R Middle for GFP reporter |
| BBC 266 | CAGCTTCGCGTCCTCGGTAGAATAAAGCAATCCGCAAGCG | P*_chb_* F Middle for GFP reporter |
| BBC 267 | CCCGGGATCCTGTGTGAAATTGAGTTGCTTTCATTTCACTAATGG | P*_chb_* R Middle for GFP reporter |
| BBC 252 | CAATTTCACACAGGATCCCGGGAGGAGGTAACGTAATGCGTAAAGGAGAAGAAC | GFP F Middle for GFP reporter |
| BBC 254 | tgtaggctggagctgcttcTTAGTTGTATAGTTCATCCATGCC | GFP R Middle for GFP reporter |
| ABD 255 | gaagcagctccagcctacaCCACAATAAGCCAGAGAGCCTTAAG | ∆lacZ F2 for Down arm |
| ABD 256 | CCCAAATACGGCAACTTGGCG | ∆lacZ R2 for Down arm |
| CKP 876 | CCTCGGGCGAGTATCAATCGcagCAAGATTTGATGCGTGCCGTG | ChiS K1025Q F |
| CKP 877 | CACGGCACGCATCAAATCTTGctgCGATTGATACTCGCCCGAGG | ChiS K1025Q R |
| CKP 878 | CGGGCGAGTATCAATCGcag | ChiS K1025Q detect F |
| CKP 879 | CAATCGAAACAAGATTTGATGCagGCCGTGTTAGTCGAAGCCATG | ChiS R1030Q F |
| CKP 880 | CATGGCTTCGACTAACACGGCctGCATCAAATCTTGTTTCGATTG | ChiS R1030Q R |
| CKP 881 | CGAAACAAGATTTGATGCag | ChiS R1030Q detect F |
| CKP 882 | GTGCCTTGAACTATTGGGAACagGTCTCAGGGCAAAGCAAGTTC | ChiS R1046Q F |
| CKP 883 | GAACTTGCTTTGCCCTGAGACctGTTCCCAATAGTTCAAGGCAC | ChiS R1046Q R |
| CKP 884 | CCTTGAACTATTGGGAACag | ChiS R1046Q detect F |
| CKP 885 | GAACGAGTCTCAGGGCAAAGCcAgTTCACGTTTGCCGAACAAAG | ChiS K1052Q F |
| CKP 886 | CTTTGTTCGGCAAACGTGAAcTgGCTTTGCCCTGAGACTCGTTC | ChiS K1052Q R |
| CKP 887 | GAGTCTCAGGGCAAAGCcAg | ChiS K1052Q detect F |
| CKP 888 | CGAACAAAGTGGCTTGTGGCagGTTTATCTTGACCGCAGCAC | ChiS R1063Q F |
| CKP 889 | GTGCTGCGGTCAAGATAAACctGCCACAAGCCACTTTGTTCG | ChiS R1063Q R |
| CKP 890 | AACAAAGTGGCTTGTGGCag | ChiS R1063Q detect F |
| CKP 891 | GTGGCGCGTTTATCTTGACCagAGCACCCTACAAACTCGTAC | ChiS R1068Q F |
| CKP 892 | GTACGAGTTTGTAGGGTGCTctGGTCAAGATAAACGCGCCAC | ChiS R1068Q R |
| CKP 893 | GGCGCGTTTATCTTGACCag | ChiS R1068Q detect F |
| CKP 894 | CCGCAGCACCCTACAAACTCagACCCTAGACAAATACTTACGAATTG | ChiS R1074Q F |
| CKP 895 | CAATTCGTAAGTATTTGTCTAGGGTctGAGTTTGTAGGGTGCTGCGG | ChiS R1074Q R |
| CKP 896 | GCAGCACCCTACAAACTCag | ChiS R1074Q detect F |
| CKP 897 | CTACAAACTCGTACCCTAGACcAgTACTTACGAATTGAGACACTG | ChiS K1078Q F |
| CKP 898 | CAGTGTCTCAATTCGTAAGTAcTgGTCTAGGGTACGAGTTTGTAG | ChiS K1078Q R |
| CKP 899 | CAAACTCGTACCCTAGACcAg | ChiS K1078Q detect F |
| CKP 900 | CTCGTACCCTAGACAAATACTTACagATTGAGACACTGCCTAAAAC | ChiS R1081Q F |
| CKP 901 | GTTTTAGGCAGTGTCTCAATctGTAAGTATTTGTCTAGGGTACGAG | ChiS R1081Q R |
| CKP 902 | CGTACCCTAGACAAATACTTACag | ChiS R1081Q detect F |
| CKP 903 | CTTACGAATTGAGACACTGCCTcAgACACCGCGTTGGCGAAC | ChiS K1087Q F |
| CKP 904 | GTTCGCCAACGCGGTGTcTgAGGCAGTGTCTCAATTCGTAAG | ChiS K1087Q R |
| CKP 905 | GAATTGAGACACTGCCTcAg | ChiS K1087Q detect F |
| CKP 906 | GACACTGCCTAAAACACCGCagTGGCGAACCGTACTGAACTC | ChiS R1090Q F |
| CKP 907 | GAGTTCAGTACGGTTCGCCActGCGGTGTTTTAGGCAGTGTC | ChiS R1090Q R |
| CKP 908 | CACTGCCTAAAACACCGCag | ChiS R1090Q detect F |
| CKP 909 | CCTAAAACACCGCGTTGGCagACCGTACTGAACTCGCTCGAC | ChiS R1092Q F |
| CKP 910 | GTCGAGCGAGTTCAGTACGGTctGCCAACGCGGTGTTTTAGG | ChiS R1092Q R |
| CKP 911 | CTAAAACACCGCGTTGGCag | ChiS R1092Q detect F |
| CKP 912 | CGACTACATTCTTGAGCATTGCcAgGAAGCAGGCCCTGAACGC | ChiS K1106Q F |
| CKP 913 | GCGTTCAGGGCCTGCTTCcTgGCAATGCTCAAGAATGTAGTCG | ChiS K1106Q R |
| CKP 914 | TACATTCTTGAGCATTGCcAg | ChiS K1106Q detect F |
| CKP 915 | CAAAGAAGCAGGCCCTGAACagACTCACATCGAAATGCAGCG | ChiS R1112Q F |
| CKP 916 | CGCTGCATTTCGATGTGAGTctGTTCAGGGCCTGCTTCTTTG | ChiS R1112Q R |
| CKP 917 | AAGAAGCAGGCCCTGAACag | ChiS R1112Q detect F |
| CKP 918 | CACTCACATCGAAATGCAGCagGATAAATTGCAAAAACTCCTGACC | ChiS R1119Q F |
| CKP 919 | GGTCAGGAGTTTTTGCAATTTATCctGCTGCATTTCGATGTGAGTG | ChiS R1119Q R |
| CKP 920 | CTCACATCGAAATGCAGCag | ChiS R1119Q detect F |
| CKP 921 | CACATCGAAATGCAGCGCGATcAgTTGCAAAAACTCCTGACCAGTG | ChiS K1121Q F |
| CKP 922 | CACTGGTCAGGAGTTTTTGCAAcTgATCGCGCTGCATTTCGATGTG | ChiS K1121Q R |
| CKP 923 | TCGAAATGCAGCGCGATcAg | ChiS K1121Q detect F |
| CKP 924 | GAAATGCAGCGCGATAAATTGCAAcAgCTCCTGACCAGTG | ChiS K1124Q F |
| CKP 925 | CACTGGTCAGGAGcTgTTGCAATTTATCGCGCTGCATTTC | ChiS K1124Q R |
| CKP 926 | AGCGCGATAAATTGCAAcAg | ChiS K1124Q detect F |
| CKP 642 | GGTATTTTGACGTTAATGACGTAGGGCATCTAGGTTTTGACGTTTTTAACGGGAATTGCA | P*_chb_* WT 60 bp EMSA probe F |
| CKP 643 | TGCAATTCCCGTTAAAAACGTCAAAACCTAGATGCCCTACGTCATTAACGTCAAAATACC | P*_chb_* WT 60 bp EMSA probe R |
| CKP 648 | GGTATTTTGAtacTAATGACGTAGGGCATCTAGGTTTTGAtacTTTTAACGGGAATTGCA | P*_chb_* Mutated 60 bp EMSA probe F |
| CKP 649 | TGCAATTCCCGTTAAAAgtaTCAAAACCTAGATGCCCTACGTCATTAgtaTCAAAATACC | P*_chb_* Mutated 60 bp EMSA probe R |
| CKP 978 | AAAATCAGGCTAGTGAGCGAG | P*_chb_* Bend probe 1 F |
| CKP 979 | TATCAATTGCAATTCCCGTTAAAAACG | P*_chb_* Bend probe 1 R |
| CKP 980 | ATATAACTCAGGCAAAGAGCC | P*_chb_* Bend probe 2 F |
| CKP 981 | AGGAGTAAGAAAACACCTAGCC | P*_chb_* Bend probe 2 R |
| CKP 982 | CAAGGCCAAATAAGTAAGTAAAC | P*_chb_* Bend probe 3 F |
| CKP 983 | GGAGTCATGAGTGGCCTGTAG | P*_chb_* Bend probe 3 R |
| CKP 984 | AACCTAGCTAAACCGTACCC | P*_chb_* Bend probe 4 F |
| CKP 985 | CCTCATCACTTTTACCCCGTC | P*_chb_* Bend probe 4 R |
| CKP 986 | TTGAATCACTTCGCGTTTTTTG | P*_chb_* Bend probe 5 F |
| CKP 987 | CATCAATTGATAAACACTCTCCAAG | P*_chb_* Bend probe 5 R |
| CKP 988 | TCACACATCAGAAGGTATTTTG | P*_chb_* Bend probe 6 F |
| CKP 989 | TTGCTTTCATTTCACTAATGGATGG | P*_chb_* Bend probe 6 R |
| CKP 990 | GACGTAGGGCATCTAGGTTTTG | P*_chb_* Bend probe 7 F |
| CKP 991 | ATATTGGCAAGCATAGCTGTTCC | P*_chb_* Bend probe 7 R |
| BBC 989 | GCATCTAGGTTTTGACGTTTTTAACG | P*_chb_* amplify for ChIP qPCR F |
| BBC 990 | AACACTCTCCAAGACCTACCTC | P*_chb_* amplify for ChIP qPCR R |
| ABD 132 | CTGTCTCAAGCCGGTTACAA | *rpoB* amplify for ChIP qPCR F |
| ABD 133 | TTTCTACCAGTGCAGAGATGC | *rpoB* amplify for ChIP qPCR R |
